# The *SbMT-2* Gene from a Halophyte Confers Abiotic Stress Tolerance and Modulates ROS Scavenging in Transgenic Tobacco

**DOI:** 10.1371/journal.pone.0111379

**Published:** 2014-10-23

**Authors:** Amit Kumar Chaturvedi, Manish Kumar Patel, Avinash Mishra, Vivekanand Tiwari, Bhavanath Jha

**Affiliations:** Discipline of Marine Biotechnology and Ecology, CSIR-Central Salt and Marine Chemicals Research Institute, Bhavnagar, Gujarat, India; National Taiwan University, Taiwan

## Abstract

Heavy metals are common pollutants of the coastal saline area and *Salicornia brachiata* an extreme halophyte is frequently exposed to various abiotic stresses including heavy metals. The *SbMT-2* gene was cloned and transformed to tobacco for the functional validation. Transgenic tobacco lines (L2, L4, L6 and L13) showed significantly enhanced salt (NaCl), osmotic (PEG) and metals (Zn^++^, Cu^++^ and Cd^++^) tolerance compared to WT plants. Transgenic lines did not show any morphological variation and had enhanced growth parameters viz. shoot length, root length, fresh weight and dry weight. High seed germination percentage, chlorophyll content, relative water content, electrolytic leakage and membrane stability index confirmed that transgenic lines performed better under salt (NaCl), osmotic (PEG) and metals (Zn^++^, Cu^++^ and Cd^++^) stress conditions compared to WT plants. Proline, H_2_O_2_ and lipid peroxidation (MDA) analyses suggested the role of *Sb*MT-2 in cellular homeostasis and H_2_O_2_ detoxification. Furthermore *in vivo* localization of H_2_O_2_ and O_2_^−^; and elevated expression of key antioxidant enzyme encoding genes, *SOD*, *POD* and *APX* evident the possible role of *SbMT-2* in ROS scavenging/detoxification mechanism. Transgenic lines showed accumulation of Cu^++^ and Cd^++^ in root while Zn^++^ in stem under stress condition. Under control (unstressed) condition, Zn^++^ was accumulated more in root but accumulation of Zn^++^ in stem under stress condition suggested that *Sb*MT-2 may involve in the selective translocation of Zn^++^ from root to stem. This observation was further supported by the up-regulation of zinc transporter encoding genes *NtZIP1* and *NtHMA-A* under metal ion stress condition. The study suggested that *SbMT-2* modulates ROS scavenging and is a potential candidate to be used for phytoremediation and imparting stress tolerance.

## Introduction

Abiotic stresses such as salinity, drought, temperature and heavy metals have significant effect on the agricultural production over the years [1]. Most often, plants may encounter these abiotic stresses simultaneously, resulting in to the substantial loss in agricultural productivity. Over the past two centuries, increased industrial and anthropogenic activities viz. mining, irrigation with sewage effluents/waste waters, use of phosphate fertilizers are the major sources of metal contamination to soil [2]–[3]. Salinity, drought and heavy metals have similar consequences ensuing oxidative stress, disturbances in ionic homeostasis and generation of reactive oxygen species (ROS) [1], [4].

Metallothioneins (MTs) are the group of polypeptides which can bind with heavy metals through their thiols group via chelation and involved in the homeostasis of essential metals (Cu and Zn) and cellular detoxification of nonessential metals (Cd and Hg) [5], [6]. Characteristics of metallothioneins are the cysteine (Cys) residue which is the basis of its classification. Based on the arrangement of Cys residues MTs are divided in two classes [7]. Plant MTs belong to Class II and further classified into four types (type 1 to 4) based on the position and allocation of cysteine residues [7]. Spatial expression of all four MT type has been reported which is localized to root, stem, leaves and developing seeds [7], [8] and show differential tolerance to different metals. Although there are several reports regarding the possible role of MTs in plants but its physiological role is still not fully conclusive because of difficulties in its isolation and stability [7], [9]. Over-expression of MTs in various model systems like Arabidopsis, tobacco, yeast and *E. coli* established its functional role in homeostasis and tolerance to Cu, Zn and Cd [10], [11], [12], [13], high salinity, drought, low temperature, heavy metal ions, abscisic acid (ABA) and ethylene [12], [14]. Besides above, MTs are reported in the inhibition of root elongation [15], fruit ripening, seed development [7] and provide disease resistance against pathogen attack [16].

Heavy metals affect the plant at cellular, biochemical and molecular level causing the oxidative stress. These toxic metals generate free radicals and reactive oxygen species (ROS) which damages the cell membrane, nucleic acids and photosynthetic pigments [17]. The equilibrium between ROS generation and quenching is prerequisite for the cell survival which is maintained by the intricate anti-oxidative system comprising of enzymatic [superoxide dismutase (SOD), catalase (CAT), peroxidase (POD)] and non-enzymatic (ascorbate, glutathione and phenolic compounds) systems [4], [12], [17], [18]. Under stress conditions the activity of this antioxidative system gets increased due to the increase of free radical formation [17].

Though MTs are known to be involved in abiotic/metal stresses and have been reported from several plant species, there are a few reports on halophytes under heavy metal stress [19]. *Salicornia brachiata* is an extreme halophyte and frequently exposed to heavy metals in coastal areas. The plant has nutritional value, unique oligosaccharide profile and requires NaCl for *in*-*vitro* regeneration [20]–[22]. The plant is considered as a model for the study of tolerance mechanism and several stress responsive genes have been isolated and characterized [13], [23]–[28]. In our previous study, *SbMT*-2 gene was isolated and physiological role was determined in *E. coli* which showed its role in the homeostasis and detoxification of Zn, Cu and Cd ions [13]. The gene (*SbMT-2*) was considered as a potential candidate to be utilized for the genetic engineering of plants for phytoremediation of heavy metals and stress tolerance. Therefore in the present study *SbMT*-2 gene was transformed into tobacco for the functional validation. Biochemical, physiological and morphological responses of transgenic plants over-expressing *SbMT-2* gene under different abiotic stresses (metals- Zn, Cu & Cd; NaCl and osmotic stresses) were studied.

## Results

### Over-expression of the *SbMT*-2 gene

**Figure 1:**
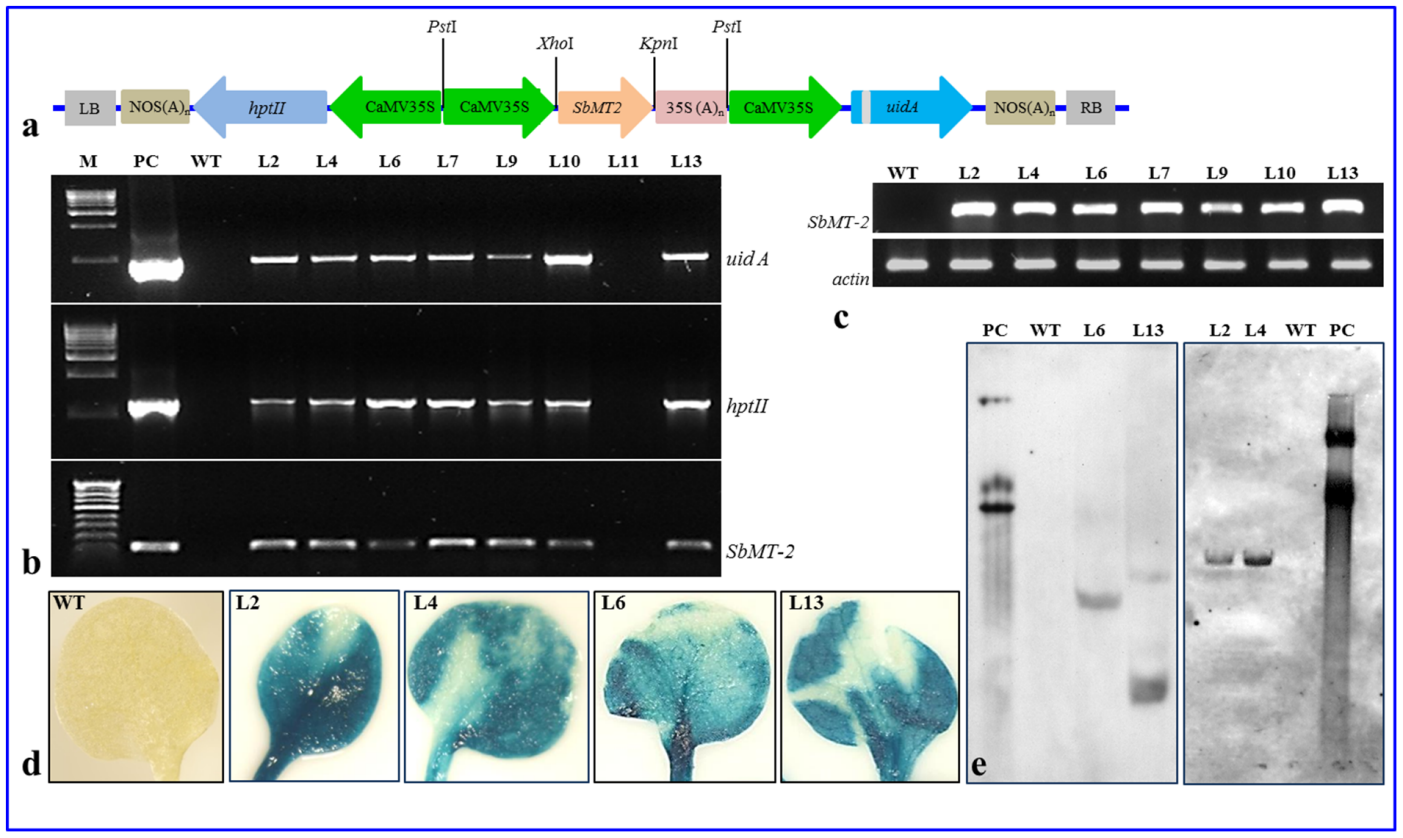
Analyses of transgenic tobacco plants. (a) Schematic map of plant expression gene construct pCAMBIA1301-*SbMT-2*. (b) Confirmation of transgenic lines by PCR amplification of *uidA*, *hptII* and *SbMT-2* genes (Lane M is molecular weight markers, Lane PC is positive control of PCR using plasmid *pCAMBIA1301-SbMT-2* as template, Lane WT is wild type control plant (non-transgenic) and Lane L is transgenic lines). (c) Over-expression of the *SbMT-2* gene in transgenic lines compared to wild-type control plants (Lane WT is wild type control plant and Lane L is transgenic lines) analyzed by semi-quantitative Rt-PCR, where *actin* was used as internal gene control. (d) Histochemical GUS staining of WT and transgenic plants. (e) Southern blot analysis (Lane PC is positive control plasmid *pCAMBIA1301-SbMT-2*, Lane WT is wild type control plant (non-transgenic) and Lane L is transgenic lines).

A plant expression vector (Figure 1a), harboring *SbMT*-2 gene driven by CaMV35S promoter was constructed and transformed to tobacco plants. Fourteen independent transgenic lines (T_0_) were raised and preliminarily screened by PCR using gene specific primers (data not shown). These lines were grown in containment facility and seeds (T_1_) were collected. T_1_ Seeds were germinated on hygromycin containing media and selected T_1_ transgenic lines were confirmed by PCR using gene specific primers (Figure 1b). Expected size of amplicon was found in all lines except L11. Based on *SbMT*-2 (Figure 1c) gene expression level, four independent T_1_ transgenic lines; L2, L4, L6 and L13 were selected for the further morpho-physio-biochemical analyses. All transgenic lines showed high *gus* and *SbMT*-2 gene expression (Figure 1c and 1d). Southern blot confirmed single and double copy gene integration to L2, L4, L6 and L13 lines, respectively (Figure 1e).

### Growth parameters in T_1_ transgenic lines

**Figure 2:**
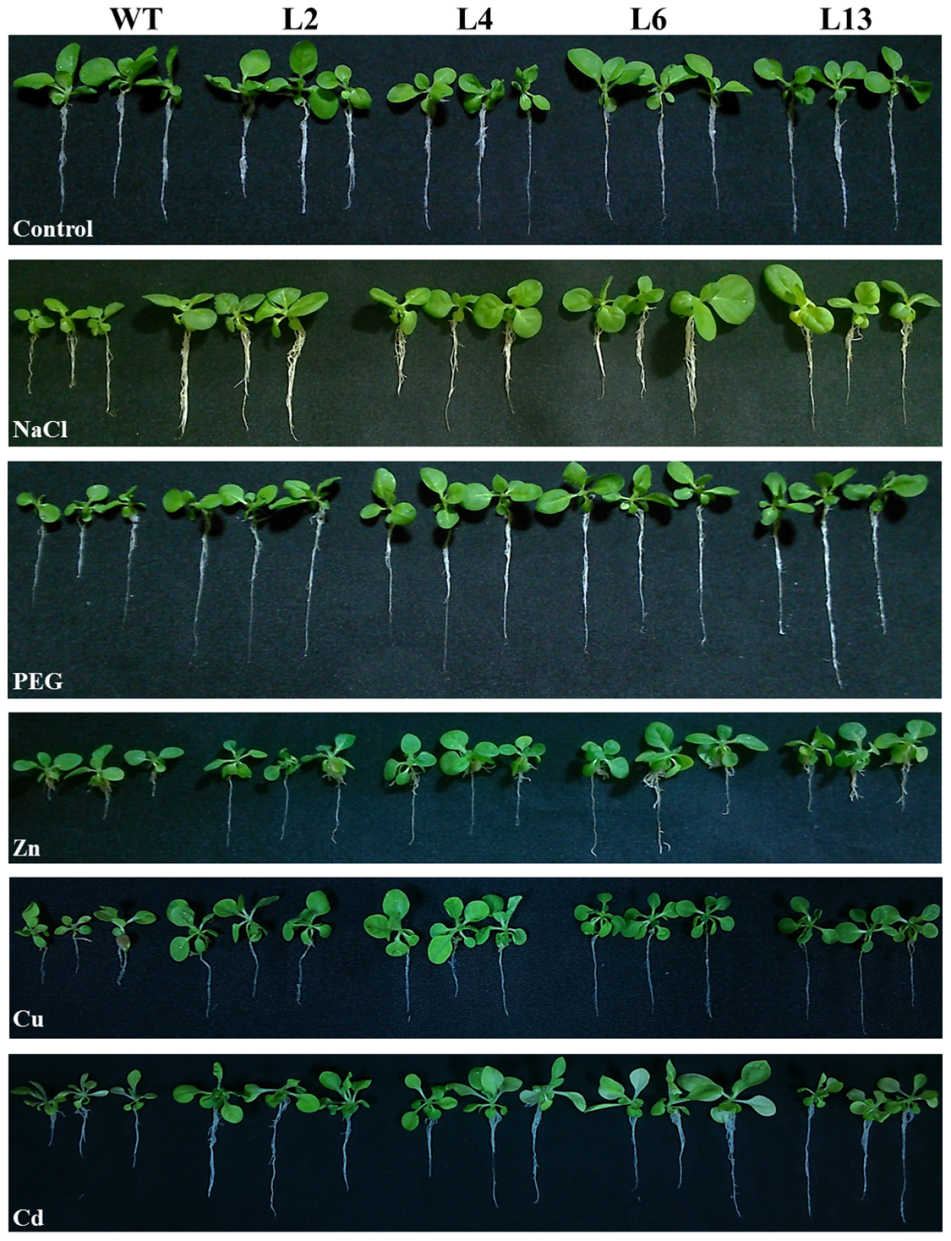
Comparative morphology of WT and T_1_ transgenic lines under different stress condition. WT and four independent T_1_ transgenic tobacco lines (L2, L4, L6 and L13) grown under control, salt (200 mM NaCl), osmotic (10% PEG), Zn (5 mM), Cu (0.2 mM) and Cd (0.2 mM) stress for 3 weeks.

**Figure 3:**
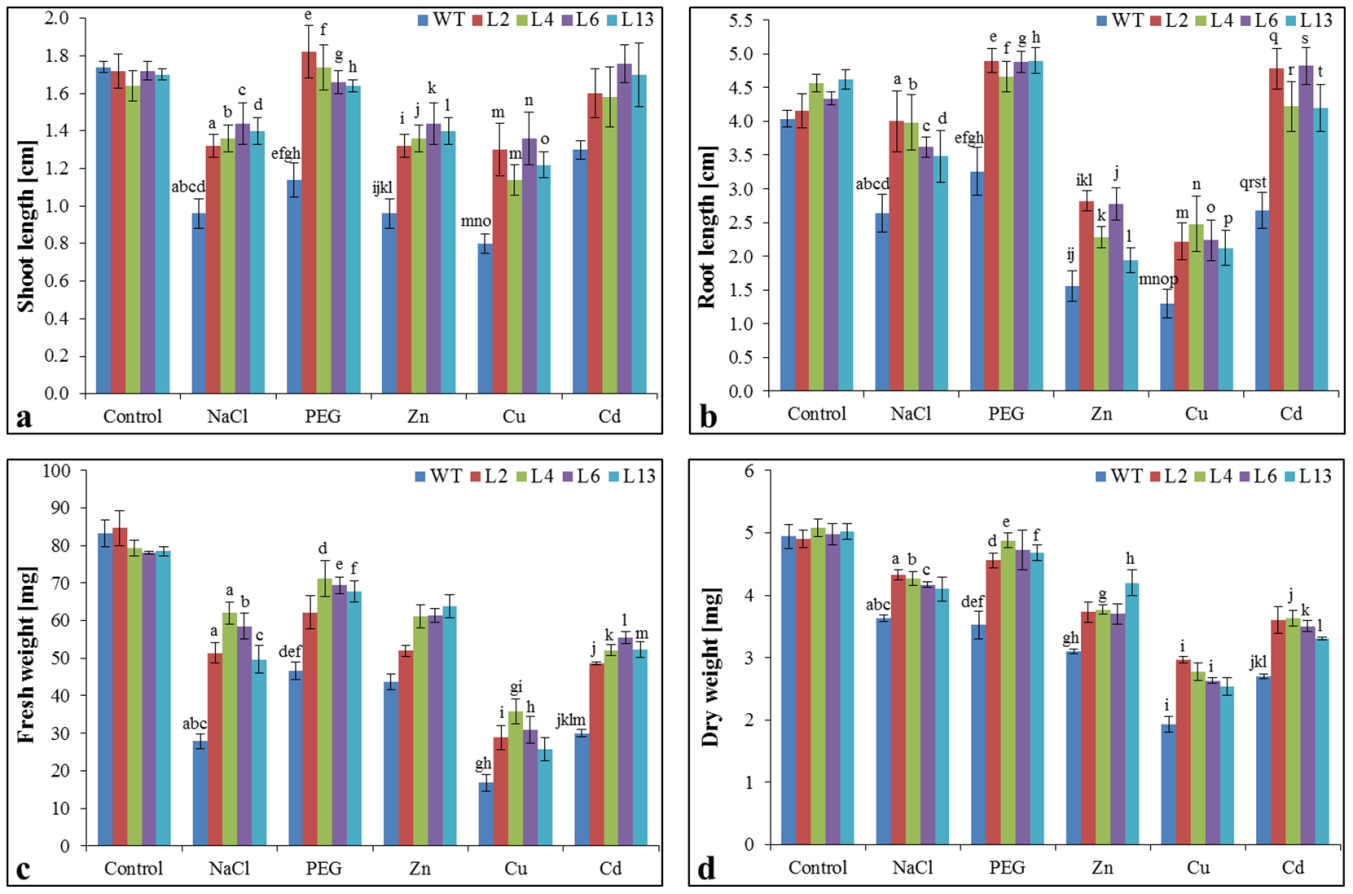
Analysis of *SbMT-2* transgenic tobacco lines under different stress condition. Comparison of (a) shoot length (cm), (b) root length (cm), (c) fresh weight (mg) and (d) dry weight (mg) of wild type (WT) and T_1_ transgenic lines (L2, L4, L6 and L13) grown under control, salt (200 mM NaCl), osmotic (10% PEG), Zn (5 mM), Cu (0.2 mM) and Cd (0.2 mM) stress for 3 weeks. Graph represents the mean ± SE (of three replicates; n = 3) followed by similar letters are significantly different according to Tukey HSD at *P<0.05*.

Transgenic lines L2, L4, L6 and L13 showed high percentage of seed germination in all stress treatments (metals- Zn, Cu and Cd; NaCl and PEG) compared to wild type (WT) plants (Figure S1). Transgenic lines grown under different stress treatments were comparatively healthier than WT plants (Figure 2) and showed enhanced growth parameters under stress conditions (Figure 3). Shoot and root lengths were found significantly higher in transgenic lines compared to WT plants in all stress treatments. Similarly, fresh and dry weights of transgenic lines were significantly higher than WT plants. Among all stress treatments studied, transgenic lines showed better growth in osmotic stress followed by metal and NaCl stress.

### Leaf senescence assay and chlorophyll content

**Figure 4:**
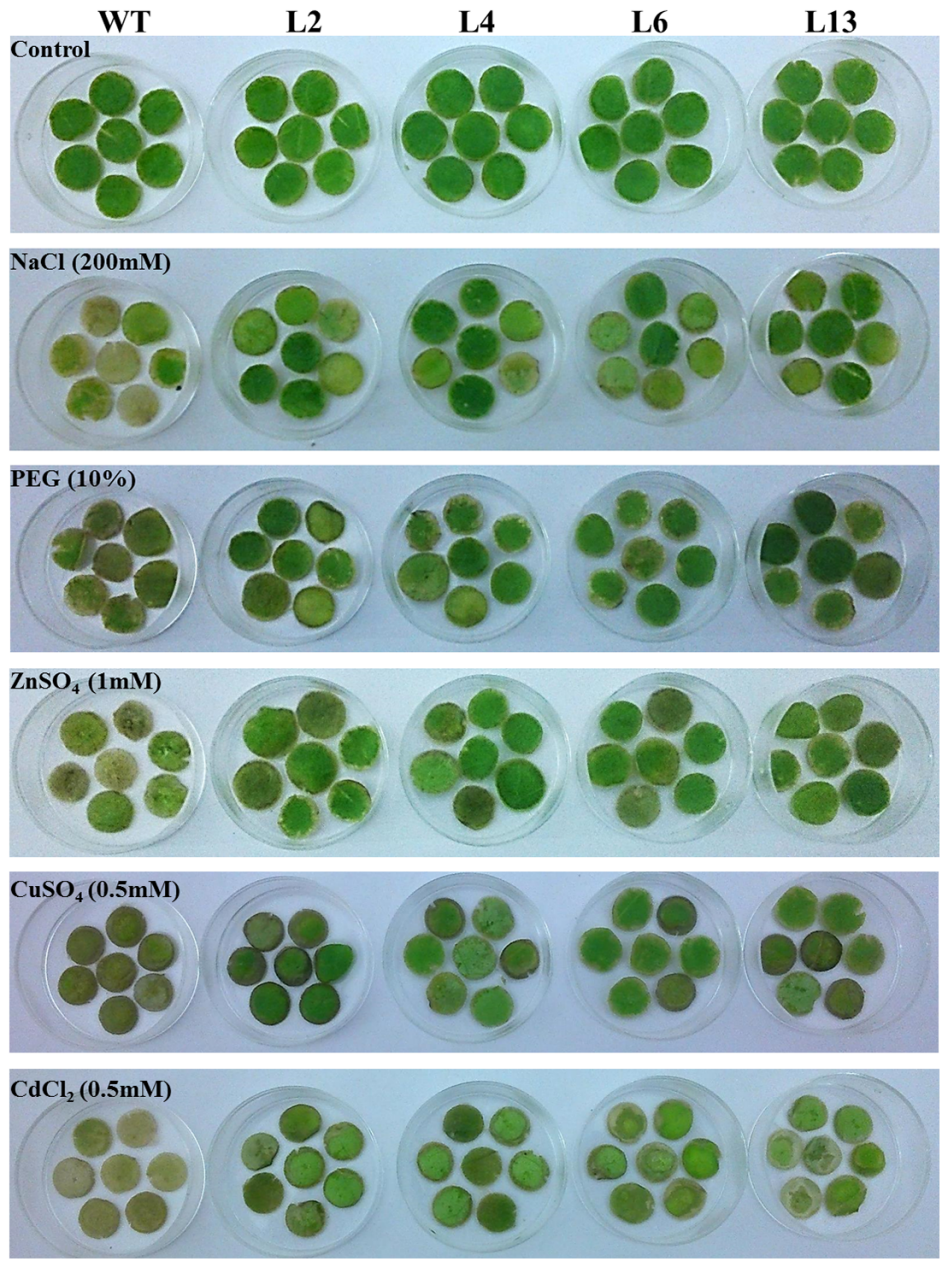
Leaf disc assay of transgenic tobacco lines (T_1_) for different stress tolerance. Leaf discs of WT, L2, L4, L6 and L13 transgenic lines respectively were floated in different stress solution for 8 days.

**Figure 5:**
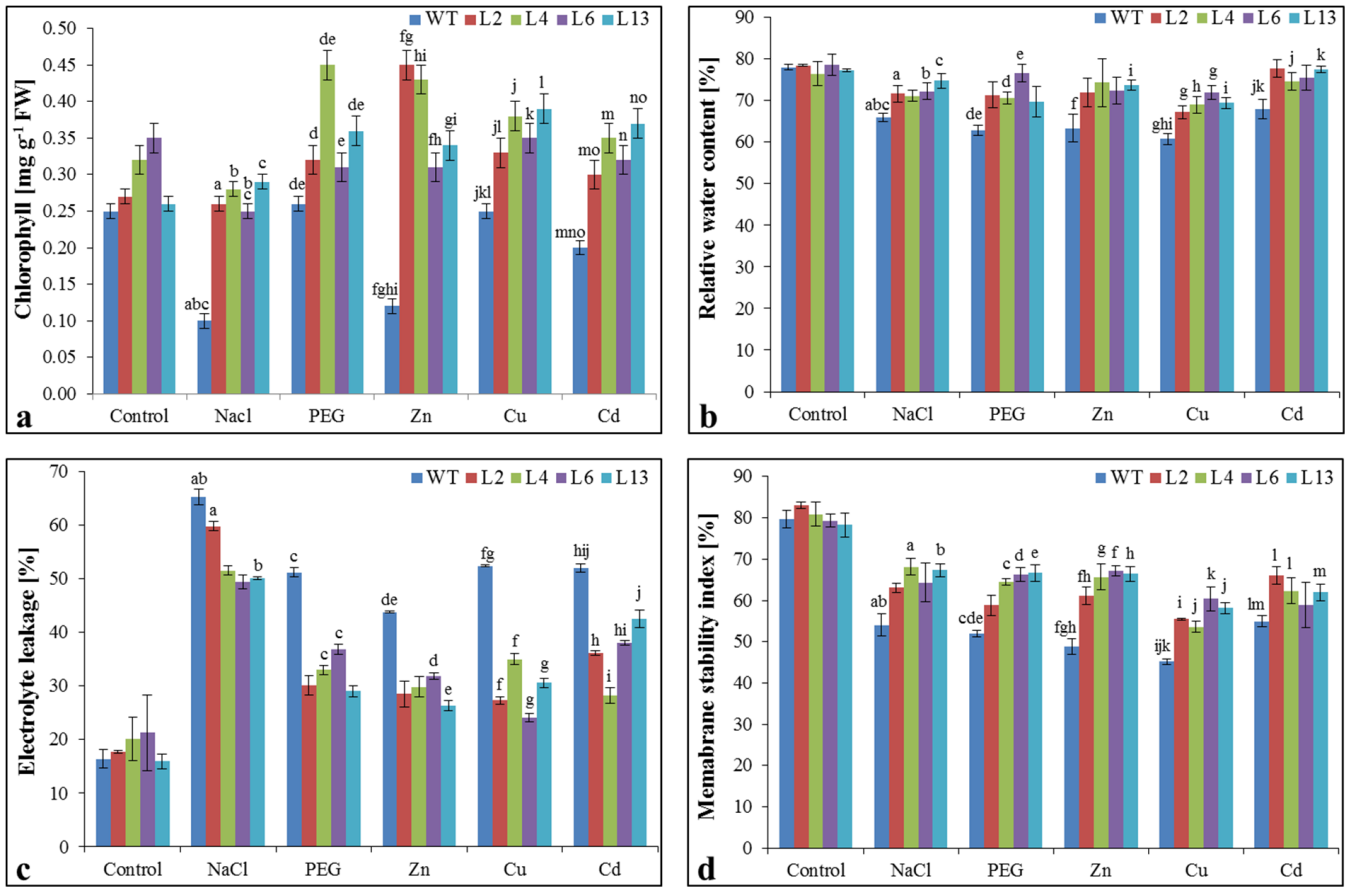
Analysis of *SbMT-2* transgenic tobacco lines under different stress condition. Comparison of (a) Chlorophyll content, (b) Relative water content, (c) Electrolyte leakage and (d) Membrane stability index of wild type (WT) and T_1_ transgenic lines (L2, L4, L6 and L13) grown in hydroponic (control) and treated with salt (200 mM NaCl), osmotic (10% PEG), Zn (1 mM), Cu (0.5 mM) and Cd (0.5 mM) stress. Graph represents the mean ± SD (of three replicates; n = 3) followed by similar letters are significantly different according to Tukey HSD at *P<0.05*.

Stress tolerance of T_1_ transgenic lines was studied by leaf disc senescence assay and chlorophyll estimation. In leaf discs assay, stress-induced necrosis resulted in the decrease of chlorophyll content was lower in the *SbMT-2* over-expressing lines compared to WT plants (Figure 4). The damage caused by stress treatments was visualized by the degree of bleaching of leaf tissues and it was evident that the transgenic plants (L2, L4, L6 and L13) had a better ability to tolerate osmotic stress followed by metal and NaCl stress (Figure 4). The chlorophyll content in the WT plants reduced significantly with stress treatments while the transgenic lines (L2, L4, L6 and L13) retained higher chlorophyll contents than WT plants (Figure 5a).

### Relative water content, Electrolyte leakage and membrane stability index

The relative water content (RWC) was found almost similar (insignificant difference) in transgenic and WT plants under unstressed condition (control), however RWC was significantly higher in transgenic lines compared to WT plants under all stress treatments (Figure 5b). Membrane permeability measured by electrolyte leakage (EL), was found significantly stable in transgenic lines, which showed reduced electrolyte leakage compared to WT plants under stress condition (Figure 5c). Similarly, membrane stability index (MSI) of transgenic lines was significantly higher compared to WT under stress treatments (Figure 5d). RWC, EL and MSI evident that transgenic lines are thriving well in stress conditions compared to WT plants.

### Localization of O_2_^−^ & H_2_O_2_ and H_2_O_2_, proline & MDA content analysis

**Figure 6:**
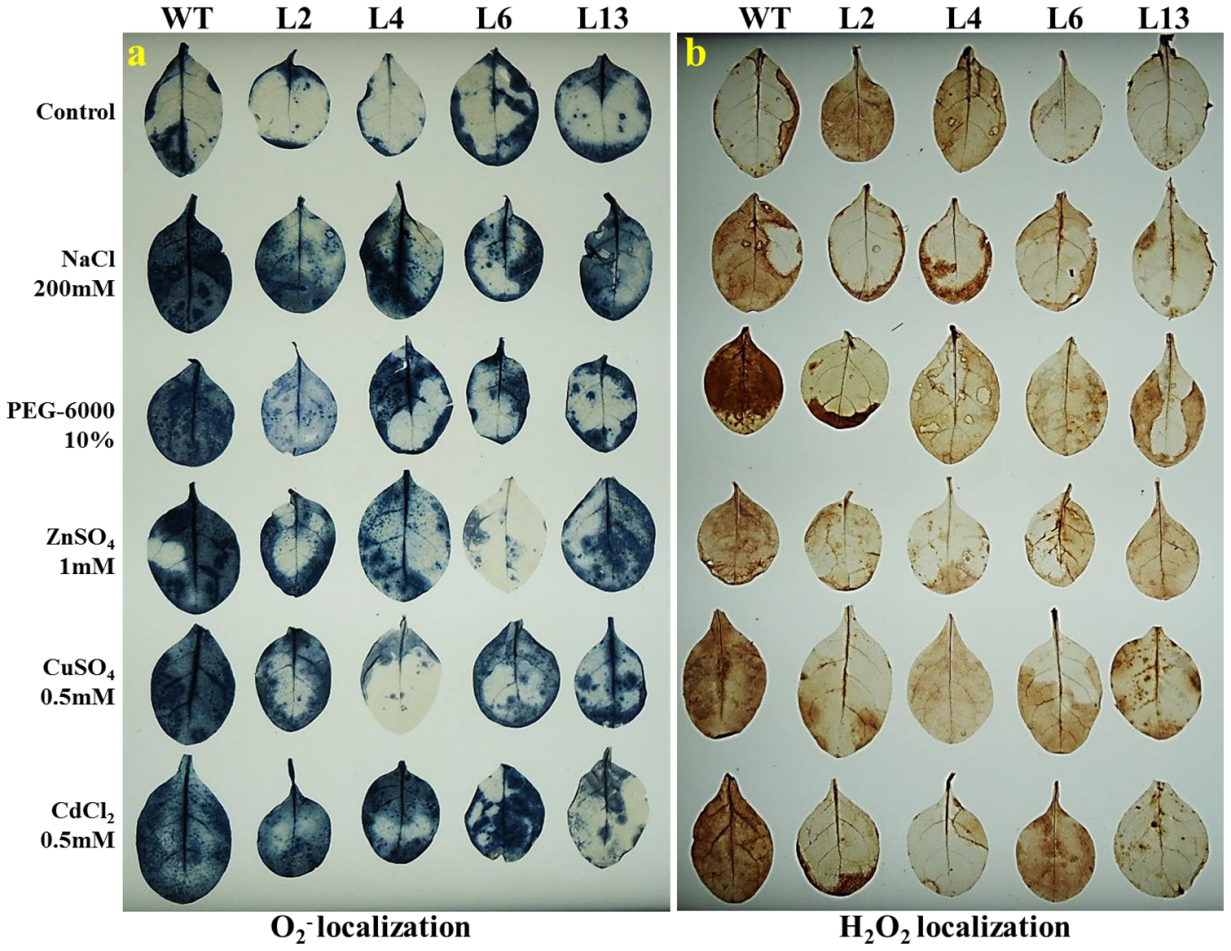
*In vivo* localization of O_2_^−^ and H_2_O_2_ in WT and transgenic lines under different stress condition. Comparison of (a) O_2_^−^ and (b) H_2_O_2_ localization in wild type (WT) and T_1_ transgenic lines (L2, L4, L6 and L13) grown in hydroponic (control) and treated with salt (200 mM NaCl), osmotic (10% PEG), Zn (1 mM), Cu (0.5 mM) and Cd (0.5 mM) stress.

**Figure 7:**
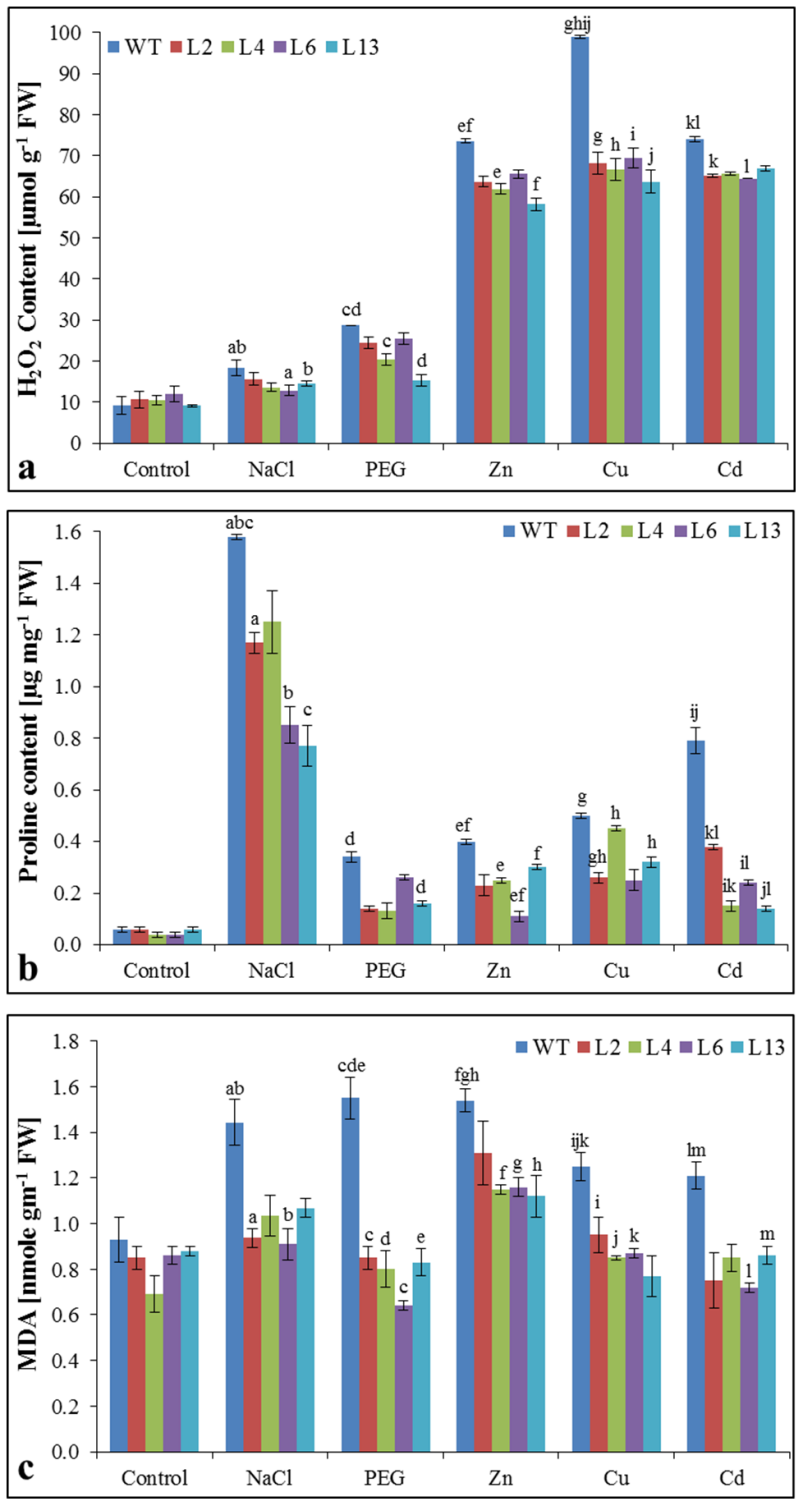
Estimation of H_2_O_2_, proline and MDA in transgenic tobacco lines under different stress condition. Comparison of (a) H_2_O_2_, (b) Proline and (c) MDA content of wild type (WT) and T_1_ transgenic lines (L2, L4, L6 and L13) grown in hydroponic (control) and treated with salt (200 mM NaCl), osmotic (10% PEG), Zn (1 mM), Cu (0.5 mM) and Cd (0.5 mM) stress. Graph represents the mean ± SD (of three replicates; n = 3) followed by similar letters are significantly different according to Tukey HSD at *P<0.05*.

Leaves of transgenic lines and WT plants, subjected to different stress treatments showed *in vivo* localization of O_2_^−^ and H_2_O_2_ (Figure 6). It was observed that leaves of WT plants showed more accumulation of O_2_^−^ and H_2_O_2_ content compared to transgenic lines under various stress conditions. Transgenic lines showed significantly lower accumulation of H_2_O_2_, proline and MDA contents under stress condition compared to WT plants (Figure 7). Lower content of H_2_O_2_ exhibited the better ROS system in transgenic lines compared to WT plants. Lower accumulation of proline and MDA revealed that transgenic lines have higher osmoprotectants and lower lipid peroxidation, respectively compared to WT plants under stress condition.

### Ion content analysis

**Figure 8:**
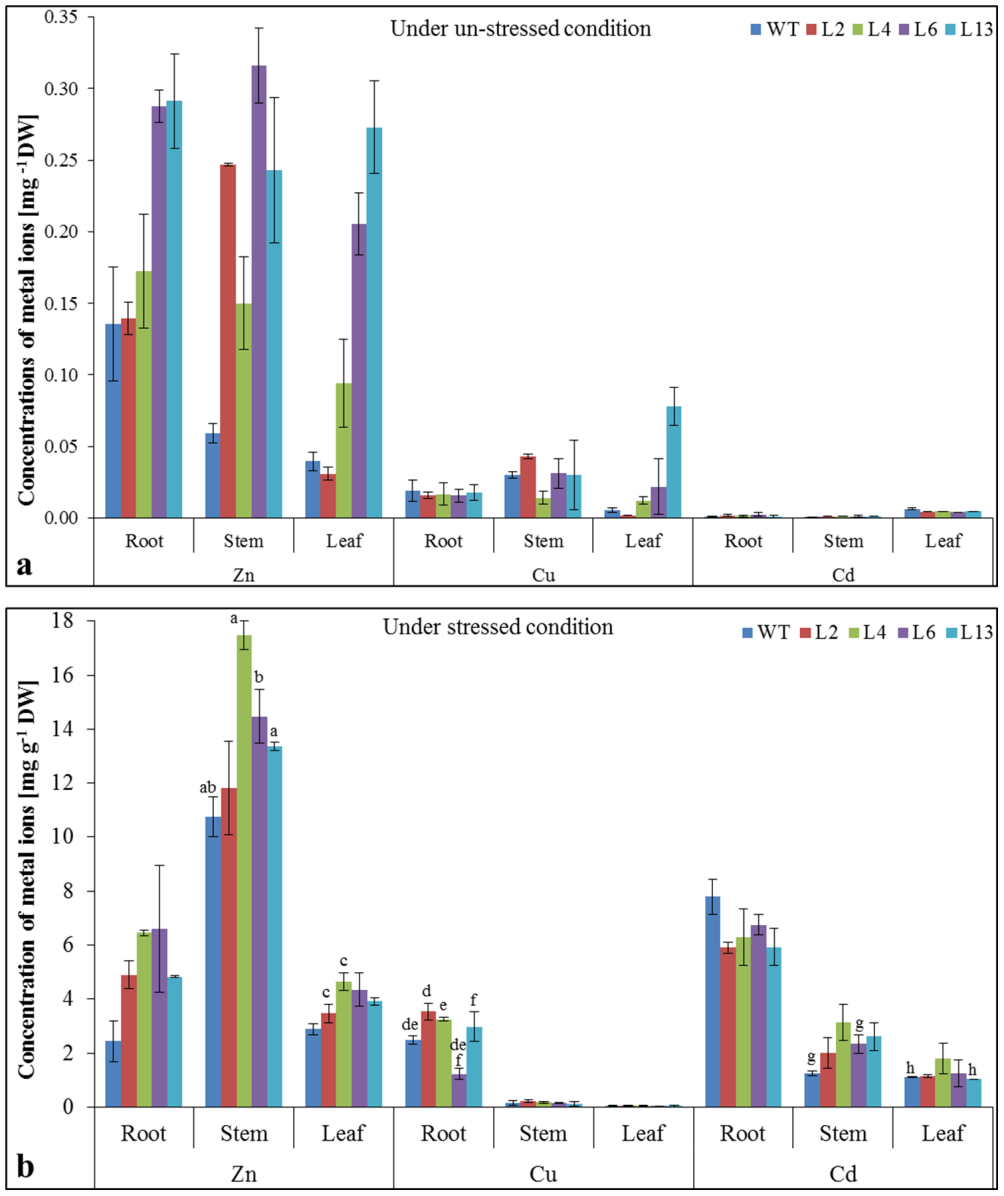
Metal ions content in transgenic tobacco lines under different stress condition. Comparison of Zn^++^, Cu^++^ and Cd^++^ content in WT and transgenic lines in root, stem and leaf under (a) control and (b) stress condition (1 mM Zn, 0.5 mM Cu and Cd). Graph represents the mean ± SD (of three replicates; n = 3) followed by similar letters are significantly different according to Tukey HSD at *P<0.05*.

Metallothioneins play a vital role in ion homeostasis and heavy metal binding. Metal ion sequestration was analyzed by ICP, which revealed high accumulation of metal ions in transgenic lines compared to WT plants (Figure 8). It was observed that metal ion contents were approximately same (insignificant difference) in WT and transgenic lines at control (unstressed) condition (Figure 8a). Under stress condition, metal ion accumulation was significantly higher in transgenic lines compared to WT plants (Figure 8b). Among different tissues, Zn accumulation was higher in shoot followed by root and leaves. However, high Cu and Cd contents were detected in roots followed by stem and leaves. Furthermore high affinity of *Sb*MT-2 was observed with Zn ions. Compared to control condition, Na^+^ contents increased in transgenic as well as WT plants under NaCl stress treatment, however K^+^ contents were decreased (Figure S2a). Similarly, transgenic lines showed higher K^+^/Na^+^ ratio compared to WT plants under NaCl stress condition (Figure S2b).

### Transcript analysis of genes encoding metal transporters and antioxidative enzymes

**Figure 9:**
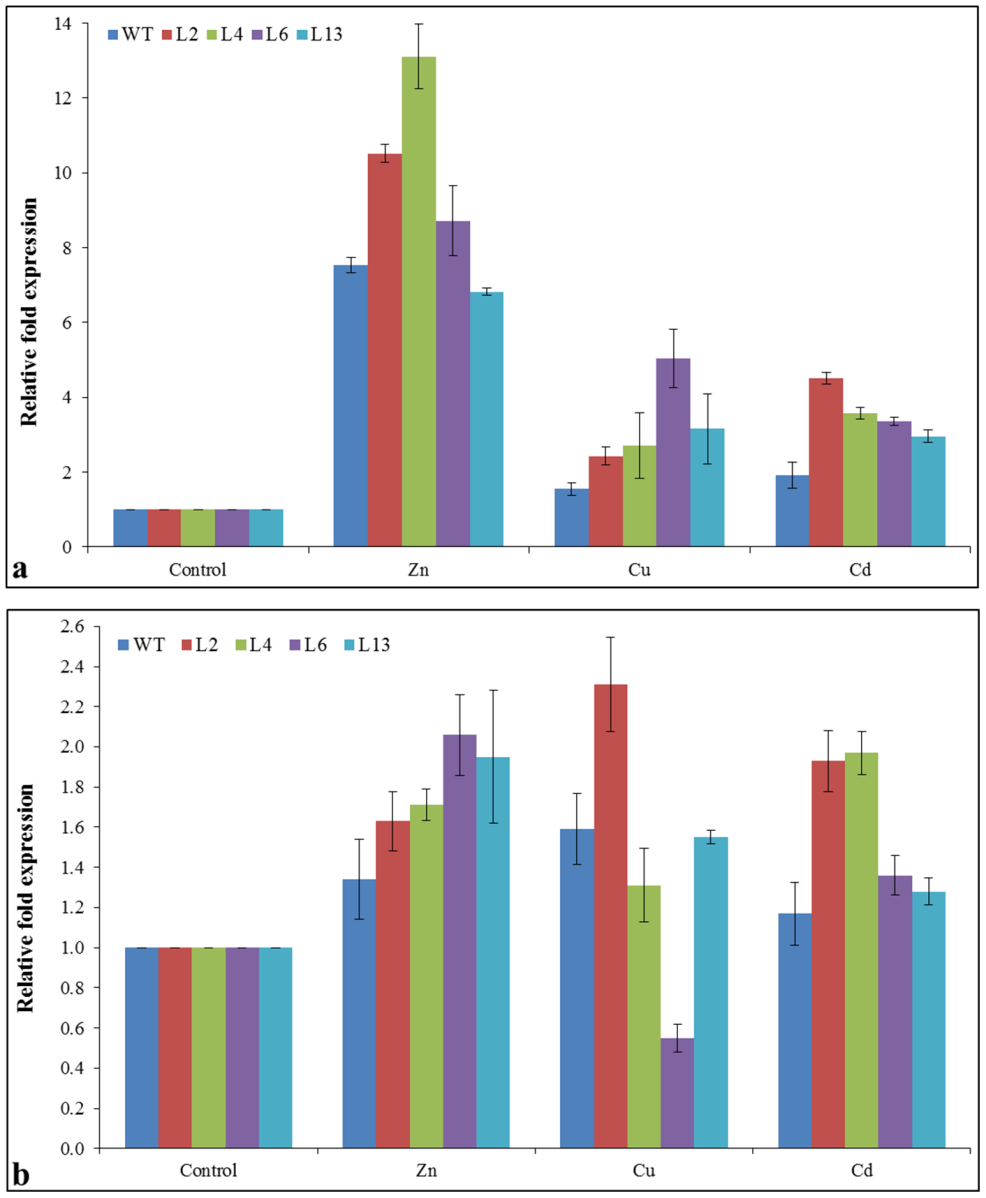
Expression analysis of zinc transporter encoding genes in transgenic tobacco lines under different metal ion stress condition. Comparison of relative fold expression of (a) *NtZIP1* and (b) *NtHMA-A* gene under Zn (1 mM), Cu (0.5 mM) and Cd (0.5 mM) stress.

Ion content analysis suggested that *Sb*MT-2 may involve in the selective translocation of Zn^++^ from root to stem. To further support this observation, expression analysis of zinc and heavy metal transporters encoding genes (*NtZIP1* and *NtHMA-A*) were studied under the metal stress (Zn, Cu and Cd) treatments. Expression of genes *NtZIP1* and *NtHMA-A* involved in heavy metal (Zn) translocation were up-regulated in transgenic lines compared to WT plants (Figure 9). Among different metal stress, the *NtZIP1* gene showed maximum up-regulation under Zn stress (Figure 9a). Transgenic lines, L2, L4, L6 and L13 showed about 10-, 13-, 8- and 6-fold expression compared to their respective control plants. However, *NtZIP1* expression was also up-regulated in WT plant. Similarly, the *NtHMA-A* gene was also up-regulated under metal stress compared to WT and control condition (Figure 9b). In contrast to other metal stress, inconsistent expression was observed among transgenic lines and WT under Cu stress treatment.

**Figure 10:**
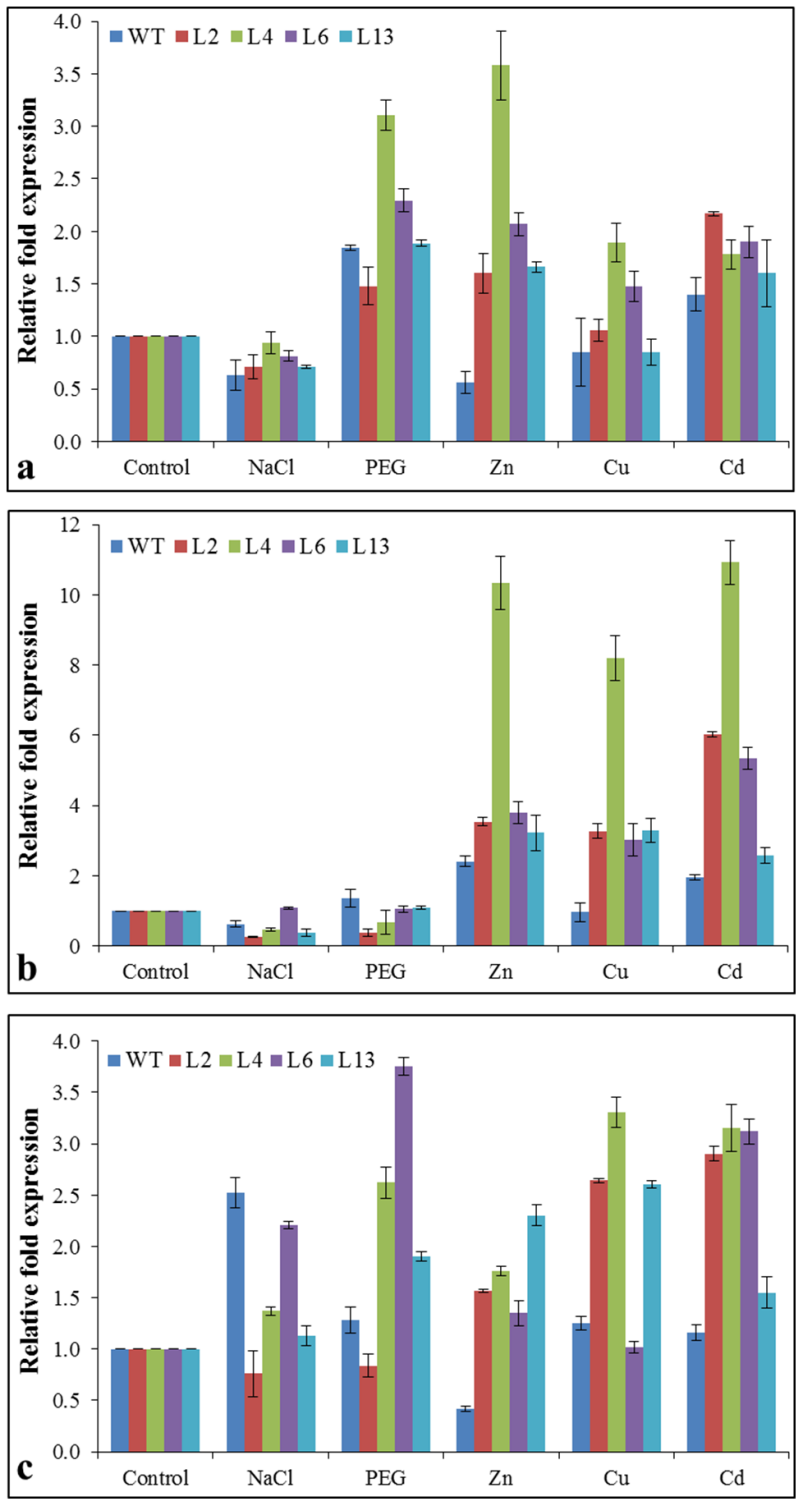
Expression analysis of antioxidant enzyme encoding genes in transgenic tobacco lines under different stress condition. Comparison of relative fold expression of (a) *NtSOD* gene, (b) *NtPOD* gene and (c) *NtAPX* gene under salt (200 mM NaCl), osmotic (10% PEG), Zn (1mM), Cu (0.5 mM) and Cd (0.5 mM) stress.

Common antioxidant enzymes involved in ROS scavenging mechanism are superoxide dismutase (SODs), peroxidase (POD) and ascorbate peroxidase (APX). Transcript analysis reveals that expression of the *NtSOD* gene increased concomitantly with stress treatments (except NaCl) in transgenic lines compared to WT plants (Figure 10a). About 3.5-fold expression was observed in L4 line under Zn and osmotic stress, while about 2-fold expression was detected under Cu and Cd stress compared to control condition (untreated plants). Remaining L2, L6 and L13 lines showed higher relative expression of *NtSOD* gene under different stress conditions compare to WT plants. Surprisingly down-regulation of gene was found under NaCl stress in transgenic as well as WT plants compared to control (untreated) plants. Relative fold expression of *NtPOD* gene was found higher in transgenic lines compared to WT plants under metal stress treatments (Figure 10b). Transgenic line L4 showed maximum gene expression about 10 -fold under Zn, Cu and Cd stress. However, *NtPOD* gene expression was down-regulated in WT and transgenic lines under NaCl and PEG stress treatments. Elevated *NtAPX* gene expression was observed in transgenic lines (except L2 under NaCl and PEG) compared to WT plants under NaCl, osmotic and metal stress conditions (Figure 10c).

## Discussion

Our previous study revealed that heterologous expression of *SbMT–2* gene not only augments zinc and copper tolerance but also increases metal ion sequestration in *E. coli* cells [13]. Further, in this study, *in planta* functional validation of *SbMT–2* gene has been elucidated. The *SbMT–2* gene was over-expressed in tobacco and its functional role was studied in different abiotic stresses *viz*. salt (200 mM NaCl), osmotic (10% PEG) and metals; Zn (1 mM), Cu (0.5 mM) and Cd (0.5 mM). Morphological variation was not observed in transgenic lines over-expressing *SbMT*-2 gene compared to WT plants (Figure 2). Transgenic lines showed higher seed germination percentage, increased root length, shoot length, fresh weight (FW), dry weight (DW) and chlorophyll content under different stress conditions compared to WT plants (Figure 3 and 5a) which reveal that *SbMT-2* leads to overcome the deleterious effect of salt, drought and metal stresses. Previously, ectopic expression of *BjMT2* gene conferred the increase in percentage seed germination, fresh weight (FW), dry weight (DW) and chlorophyll content in *Arabidopsis* under Cu and Cd stress [15]. Similar results were also observed under salt, drought, Zn and Cu stress in transgenic tobacco plants over-expressing clustered *OsMT1e-P* metallothionein gene [29].

Salt, drought and heavy metal stress are multigenic in nature, causing osmotic stress and thus create physiological drought conditions for plants. Transgenic lines displayed increase in relative water content under stress (Figure 5b) which indicates that *SbMT-2* gene may help in water retention to counteract the osmotic shock. Abiotic stress causes the perturbation in metabolic balance of the cell, resulting in the enhanced production of ROS, which in-turn damages cell membranes, nucleic acids and chloroplast pigments [15], [30]. Electrolyte leakage (EL) and membrane stability index (MSI) is the indicator of cell membrane stability. In the present study electrolyte leakage was lower in transgenic lines while MSI was higher under stress condition (Figure 5c-d) which suggests the role of *SbMT-2* gene in cell membrane protection. It was further supported by lipid peroxidation analysis, H_2_O_2_ and proline quantification, in which high MDA, H_2_O_2_ and proline contents were found in WT plants compared to transgenic lines under stress condition (Figure 7). These result evident the possible role of *Sb*MT-2 in ROS scavenging/detoxification and maintaining the cellular homeostasis during stress condition. Furthermore, *in vivo* localization of H_2_O_2_ and O_2_^-^ under different abiotic stress (Figure 6), confirmed the role of *SbMT*-2 gene in ROS scavenging.

Although role of metallothionein genes in ROS scavenging and detoxification have studied [12], [14], [16], [29], [31] but the molecular mechanism of ROS detoxification/scavenging is still unknown. ROS, being a signaling molecule play an important role in development and regulation of different metabolic processes whereas ROS toxicity resulted into the oxidative damage to the cell membrane and its components [30]. Plant cell maintains a stringent regulation over its production and scavenging. In *Arabidopsis* about 150 genes are involved in the homeostasis of ROS which comprised of ROS scavenging enzymes and ROS producing proteins [30]. Superoxide dismutase (SOD), peroxidase (POD) and ascorbate peroxidase (APX) are important ROS scavenging enzymes activated under different stress conditions to maintain the ROS homeostasis. Expression of genes encoding these ROS scavenging enzymes was studied in transgenic lines and compared with WT plants (Figure 10) to confirm the role of the *SbMT-2* gene in ROS scavenging and detoxification. Higher relative fold expression of *NtSOD* and *NtPOD* genes were found in transgenic lines under stress conditions, however similar result was also observed with the *NtAPX* gene except under salt stress. Moreover, high expression was detected in metal stress compared to salt and osmotic stress, which may be because of availability of cofactors of the enzymes.

Metallothioniens are involved in essential-metal homeostasis and impart protection against heavy metal toxicity by sequestration [6], [9]. Plants maintain a high K^+^/Na^+^ ratio to combat with the deleterious effect of salinity. In present study transgenic lines over-expressing *SbMT-2* gene showed a high K^+^/Na^+^ ratio under stress compared to WT plants and control condition (Figure S2). It provides further evidence that *Sb*MT-2 may have role in ionic homeostasis and detoxification of H_2_O_2_ and thus impart salt tolerance. Transgenic lines showed accumulation of Cu^++^ and Cd^++^ in root compared to stem and leaves under stress condition. However, the *SbMT-2* gene over-expression leads to the accumulation of Zn^++^ in stem compared to control condition where it was accumulated more in roots (Figure 8). Zinc ion accumulation and tolerance varies among plant species and depends on MT type. The result exhibits *Sb*MT-2 may involve in the selective translocation of Zn^++^ from root to stem. Furthermore, the result also suggests that *Sb*MT-2 is involved in Cu and Cd binding and accumulation rather than translocation, as observed with Zn ions. Previously, it was reported that *Sb*MT2 protein exhibited high binding affinity and sequestration for Zn^++^ compared to Cu and Cd ions [13].

In order to support this observation, expression of type 1B heavy metal–transporting P-type ATPases (P_1B_ ATPase) transporter and zinc specific transporter encoding gene *NtHMA-A* and *NtZIP1* were analyzed under metal stress treatments (Figure 9). Up-regulation of genes in transgenic lines under metal stress especially, zinc compared to control and WT plants provides a supporting evidence that expression of *SbMT-2* gene may influence metal transporters and thus translocation of Zn was observed in the study. The qPCR analysis revealed higher up-regulation of these genes in transgenic lines compared to WT plants (about 6 to 10 fold up-regulation of *NtZIP1* gene in transgenic lines compared to about 2.5 fold of WT plants) under metal stress condition and it might be due to introgression of *SbMT*-2 gene. Furthermore, higher down-regulation of these genes (*NtZIP1*and *NtHMA-A*) in transgenic plants compared to WT was observed under de-stress treatments, performed by re-culturing the plants in un-stressed conditions (Table S1). Results further suggest that the *SbMT2* gene may influence the expression of these genes.

It was observed that expression of MT type 2 gene *PsMT(A1)* of *Pisum sativum* enhanced metal tolerance in white poplar and accumulation of zinc and copper (in leaves and roots) respectively [11]. Expression of seed specific MT gene *MT4a* in *Arabidopsis* increased Cu^++^ accumulation but did not show any response under Zn stress [10]. The *OsMT1a*, a type 1 metallothionein gene, involved in the Zn^++^ accumulation and thus provides tolerance to the transgenic rice [12]. *Elsholtzia haichowensis* metallothionein 1 (*Eh*MT1) over-expression in tobacco plants enhances copper tolerance and accumulation in root cytoplasm [32] however, expression of *BjMT2* gene in *A. thaliana* showed copper and cadmium tolerance [15]. Sequestration, translocation and thereby accumulation are the important mechanism used by plants for the phytoextraction [33] and therefore *SbMT-2* gene may be utilized for the phytoremediation.

## Conclusion

**Figure 11:**
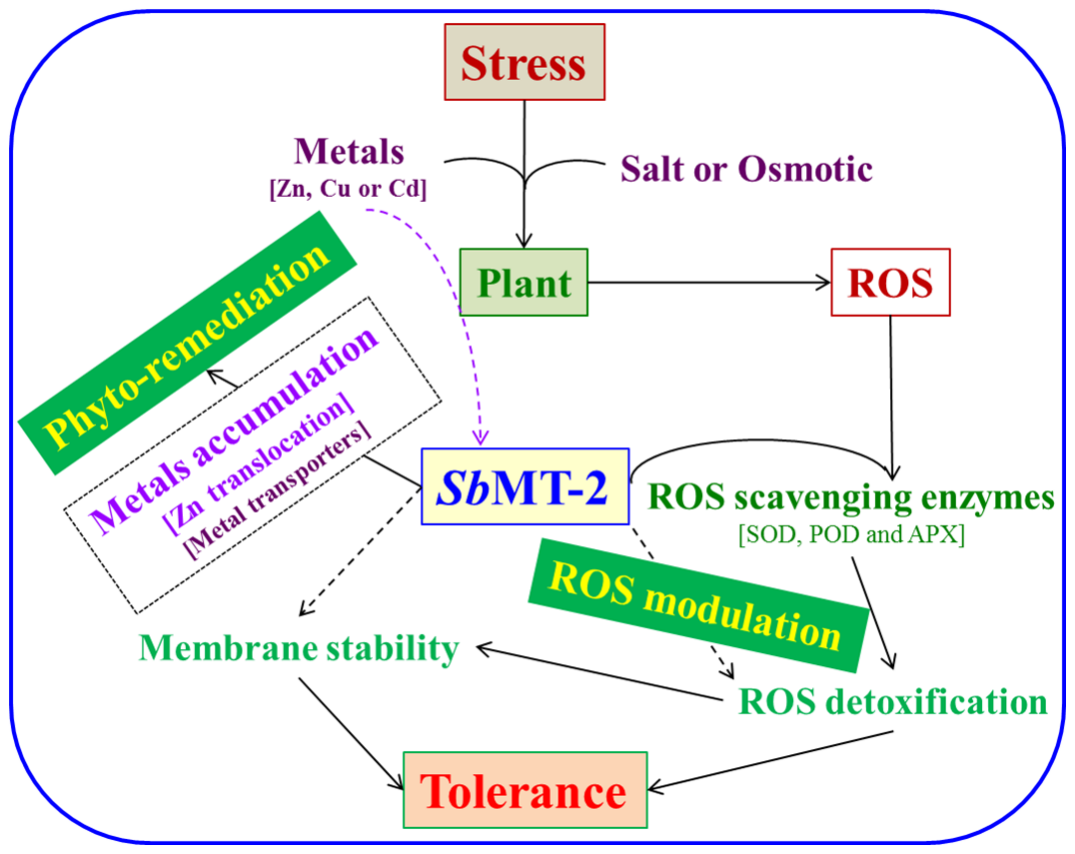
A hypothetical model for the role of *Sb*MT-2 in phyto-remediation and ROS modulation under abiotic stress.

In conclusion, the present study provides an useful insight that *Sb*MT-2 may involve in maintaining the cellular homeostasis by modulating ROS scavenging/detoxification during stress conditions and thus impart tolerance to salt and osmotic stress. It was observed that *Sb*MT-2 provides protection against heavy metal toxicity by metal ions accumulation and may be involved in the selective translocation of Zn^++^ from roots to stem (Figure 11). It is speculated that *SbMT-2* gene is a potential candidate for introgression to crop plants for imparting stress tolerance and phytoremediation.

## Methods

### Construction of plant transformation vector and tobacco transformation

The *SbMT-2* cDNA was amplified using forward (MT-2F: 5′- CTCGAGATGTCTCTTGCTTGTGGTGGTAAC-3′) and reverse MT-2R: 5′- GGTACCTCATTTTGCAAGTGCAAGGGTTG -3′) primers containing *Xho*I and *Kpn*I sites respectively. The amplicon was digested with *Xho*I/*Kpn*I and cloned in pRT101 vector [34]. Thereafter, the gene cassette “CaMV35S-*SbMT-2*” was excised with *Pst*I and cloned into the pCAMBIA1301 vector. The resulting vector was mobilised into *Agrobacterium tumefaciens* (EHA105) and further transformed to tobacco (*Nicotiana tabacum* cv. xanthii) plants according to the standard protocol [35].

### Confirmation of transgenic lines and expression of transgene

Total genomic DNA was isolated from leaves of transgenic and wild type (WT; control/untransformed) tobacco plants (T_1_) using Qiagen DNeasy plant mini kit and quantified by Nanodrop Spectrophotometer (ND1000, Wilmington, USA). The integration of transgene in different lines was confirmed by PCR analyses using the *SbMT-2* gene, reporter gene *gus* and selection marker gene *hptII* specific primers (Table 1). Presence and expression of the *SbMT-2* gene in selected lines (L2, L4, L6 and L13) were confirmed by Southern blot and semi-quantitative Rt-PCR, respectively.

**Table 1:** Primer sets used for the confirmation and transcript analysis of transgenic lines.

Genes	Primer sequences (5′ to 3′)	PCR conditions
*uidA*	F: GATCGCGAAAACTGTGGAAT	95°C, 5min; 34 Cycles: 95°C, 1min; 60°C, 1min; 72°C, 2 min; Extension: 72°C, 7 min
	R: TGAGCGTCGCAGAACATTAC	
*hptII*	F: TTCTTTGCCCTCGGACGAGTG	95°C, 5min; 34 Cycles: 95°C, 1min; 55°C, 1min; 72°C, 2 min; Extension: 72°C, 7 min
	R: ACAGCGTCTCCGACCTGATG	
*SbMT-2*	F: AGGTTGCAAGATGTTCC TG	95°C, 5min; 35 Cycles: 95°C, 30sec; 60°C, 45sec; 72°C, min; Melt curve: 60–95°C
	R: TCCATCGTTCTCAGCTGCTAC	with 0.5°C/cycle increment
*Actin*	F: CGTTTGGATCTTGCTGGTCGT	
	R: CAGCAATGCCAGGGAACATAG	
*NtZIP1*	F: TGGTGGCTCAGTCTGGAGAT	
	R: CGAAGGAGCTCAGAACTGGAA	
*NtHMA-A*	F: ACAAAGTGCTCGGACACCAA	
	R: CTTCTCGGTTGCAGAGTCCT	
*NtSOD*	F: AGCTACATGACGCCATTTCC	
	R: CCCTGTAAAGCAGCACCTTC	
*NtPOD*	F: CTTGGAACACGACGTTCCTT	
	R: TCGCTATCGCCATTCTTTCT	
*NtAPX*	F: CAAATGTAAGAGGAAACTCAGAGGA	
	R: CAGCCTTGAGCCTCATGGTACCG	

Genomic DNA (20 µg) from WT and transgenic lines L2, L4, L6 and L13 was digested with *Hind*III, separated on agarose (0.7%) by electrophoresis and transferred to a Hybond N^+^ membrane (Amersham Pharmacia, UK) using alkaline transfer buffer (0.4 N NaOH with 1 M NaCl). DNA blot was hybridized with PCR-generated probe for the *uidA* gene labeled with DIG-11-dUTP following pre-hybridization and hybridization carried out at 42°C overnight in DIG EasyHyb buffer solution [36]–[39]. The hybridized membrane was detected by using CDP-Star chemiluminescent as substrate, following manufacturer user guide (Roche, Germany) and signals were visualized on X-ray film after 30 min.

Total RNA was extracted from transgenic tobacco (T_1_) and wild-type plants using Qiagen RNeasy plant mini kit and the cDNA was made by reverse transcription with Superscript II RT (Invitrogen, USA). The actin gene was used as an internal control and both genes (*SbMT-2* and actin) were amplified using gene specific primers (Table 1). Histochemical GUS assay was performed with leaves as described by Jefferson [40].

### Analyses of transgenic plants under different abiotic stress

Transgenic and wild type tobacco lines were maintained under controlled containment facility. Further morphological and physio-biochemical analyses were performed with T_1_ transgenic lines under different abiotic stress treatments.

### Growth parameters

Seeds of transgenic lines (L2, L4, L6 and L13) and WT plants were germinated on MS medium supplemented with 200 mM NaCl, 10% PEG 6000, 5 mM ZnSO_4_, 0.2 mM CuSO_4_ or 0.2 mM CdCl_2_ under culture room conditions and the percentage of seed germination was calculated after 20 days.

Different growth parameters were measured under same stress conditions using T_1_ seedlings. Seeds of transgenic lines were germinated on the MS media supplemented with 20 mg/l hygromycin, while seeds of WT plant were germinated on MS media only. WT and hygromycin positive T_1_ seedlings were transferred to MS medium supplemented with 200 mM NaCl, 10% PEG, 5 mM ZnSO_4_, 0.2 mM CuSO_4_ or 0.2 mM CdCl_2_ after eight days and grown for further 21 days. Growth parameters; shoot length, root length, fresh weight and dry weight were recorded and compared with wild type plants.

### Leaf senescence assay and chlorophyll estimation

Leaf discs from 30 days old WT and transgenic plants (L2, L4, L6 and L13) were used for the stress tolerance assay. Healthy leaves of similar age were detached and leaf discs (of 5 mm in diameter) were punched out. About 8 discs of each plant were floated in 5 ml ½ Hoagland media (control) supplemented with 200 mM NaCl, 10% PEG, 1 mM ZnSO_4_, 0.5 mM CuSO_4_ or 0.5 mM CdCl_2_ for 7 days. The effect of these treatments on leaf discs were assessed by observing phenotypic changes.

Leaf discs (control and treated) were further subjected to chlorophyll isolation. Leaf discs were thoroughly homogenized in chilled N, N-dimethylformamide (DMF) at 4°C and thereafter centrifuged at 10,000 g for 10 min. Supernatant was aspirated out and O.D. was recorded at 664 and 647 nm. Total chlorophyll content was calculated per gram fresh weight of tissue according to Porra et al. [41].

### Relative water content, Electrolyte leakage and Membrane stability index

WT and T_1_ seedlings (L2, L4, L6 and L13) were transferred to ½ Hoagland hydroponics culture and maintained for 20 days. Healthy young plants of same age and size were collected for each treatment (24 h for 200 mM NaCl and 0.5 mM CuSO_4_; 12 h for 10% PEG, 1 mM ZnSO_4_ and 0.5 mM CdCl_2_).

About 100 mg (FW) leaves of control and treated plants were submerged into the deionised water and after 12 h turgid weight (TW) were recorded. Samples were further kept at 80°C for 48 h to record the dry weight (DW). The relative water content was calculated as: *RWC (%)  =  (FW-DW/TW-DW) ×100.*

Collected leaves were washed thoroughly with deionized water to remove surface-adhered electrolytes. Samples were kept in closed vials containing 10 ml deionised water and incubated at 25°C on a rotary shaker for 24 h. Subsequently, the electrical conductivity (EC) of the solution (L_t_) was determined using conductivity meter (SevenEasy, Mettler Toledo AG 8603, Switzerland). Samples were autoclaved at 120°C for 20 min, cooled up to 25°C and electrical conductivity (L_0_) was determined. The electrolyte leakage was determined: *Electrolyte leakage (%)  =  (L_t_/L_0_) ×100.*

Healthy young leaves of stress-treated plants were taken and membrane stability index (MSI) was determined [42]. Leaves (200 mg) were kept in close vials containing 10 ml deionized water. A set of vials was incubated at 40°C for 30 min while second set of vials was incubated at 100°C for 10 min. Electrical conductivity was recorded for both sets (L1 for 40°C while L2 for 100°C) and MSI was calculated using the formula as: *MSI  =  [1- (L1/L2)] ×100.*

### *In vivo* localization of O_2_^−^ and H_2_O_2_

Histochemical staining was performed for the *in vivo* detection of O_2_^−^ and H_2_O_2_ using nitro-blue tetrazolium (NBT) and 3, 3- diaminobenzidine (DAB), respectively [43]. The presence of O_2_^−^ in transgenic and WT leaves exposed to different stresses (as described above) was detected by immersing the leaf samples in NBT solution (1 mg ml^−1^ in 10 mM phosphate buffer; pH 7.8) at room temperature for 2 h and then illuminatedfor 12 h in light until blue spots appeared. For the localization of H_2_O_2_, treated leaves were incubated in DAB solution (1 mg ml^−1^ in 10 mM phosphate buffer; pH 3.8) at room temperature for 6 h in dark, thereafter exposed to the light until brown spots were appeared. Leaf samples were treated with absolute ethanol (for bleaching chlorophyll contents) before the documentation.

### Quantification of H_2_O_2_, proline and lipid peroxidation

The H_2_O_2_ and free proline content in leaf samples (WT and transgenic lines under different stress conditions) were measured [44], [45]. Proline and H_2_O_2_ levels were calculated by the standard curve, prepared against known concentration of proline or H_2_O_2_ measured at 520 and 560 nm absorbance respectively. Lipid peroxidation was estimated by determining the concentration of malondialdehyde (MDA) produced by thiobarbituric acid (TBA) reaction [46]. Leaf samples were extracted in 2 ml 0.1% trichloroacetic acid (TCA) and 0.5 ml extract was reacted with 2.0 ml of TBA reagent followed by boiling at 95°C for 30 min. Samples were cooled at ice, centrifuged at 10000 g for 5 min and absorbance of the supernatants was measured at 440 nm, 532 nm and 600 nm.

### Ion content analysis

For ion content analysis, tissues (root, shoot and leaves) from 4-week-old plants grown in hydroponic medium and treated with 200 mM NaCl, 10% PEG 6000, 1 mM ZnSO_4_, 0.5 mM CuSO_4_ or 0.5 mM CdCl_2_ were washed with deionised water, dried in hot air oven for 48 h at 70°C and digested with 4 ml perchloric acid-nitric acid solution (3:1). The solution was heated to dry and further diluted to 25 ml with deionised water. Ion contents were measured by inductively coupled plasma optical emission spectrometer (Optima2000DV, PerkinElmer, Germany).

### Transcriptional regulation of metal transporters and antioxidative enzymes

Total RNA was isolated from the stressed and control plants by using RNeasy plant mini kit (Qiagen) following the manufacturer's instructions and quantified with Nanodrop spectrophotometer (NanoDrop, USA). The cDNA was prepared using 2 µg of total RNA with a SuperScript RT III first-strand cDNA synthesis kit (Invitrogen, USA). The expression pattern of genes; *NtZIP1*, *NtHMA-A,* encoding metal transporters [47] and *NtSOD*, *NtPOX* and *NtAPX*, encoding antioxidant enzymes [48] which involved in heavy metal transportation and ROS scavenging, respectively were analyzed under different stress by using gene-specific primer pairs (Table 1), while the gene actin was used as an internal control. The quantitative real time PCR (qRT-PCR) was performed in a Bio-Rad IQ5 detection system (Bio-Rad, USA) with QuantiFast Kit (Qiagen, USA). Fold expression was calculated by the method described by Livak and Schmittgen [49] and specificity of qRT-PCR was monitored by melt curve analysis.

### Statistical analysis

Each experiment was carried out in three replicates. All data was expressed as mean ± SD and subjected to analysis of variance (ANOVA) to determine the significance of difference between the means of WT and transgenic plants of each treatment group. A Tukey HSD multiple comparison of mean test was used, significant differences were considered at *P<0.05* and indicated by similar letters.

## Supporting Information

Figure S1 **Percentage of seed germination under different stress condition.** Seeds of WT and transgenic lines were germinated on MS media supplemented with 200 mM NaCl, 10% PEG, 5 mM Zn, 0.2 mM Cu or 0.2 mM Cd. Graph represents the mean ± SD (of three replicates; n = 3) followed by similar letters are significantly different according to Tukey HSD at *P<0.05*.
(TIF)

Figure S2 **Sodium and potassium ion content in transgenic tobacco lines under different stress condition.** Comparison of (a) Na^+^ and K^+^ content and (b) K^+^/Na^+^ ratio in WT and transgenic lines in root, stem and leaf under stressed (200 mM NaCl) and un-stressed conditions. Graph represents the mean ± SD (of three replicates; n = 3) followed by similar letters are significantly different according to Tukey HSD at *P<0.05*.
(TIF)

Table S1 **Comparison of relative fold expression (down–regulation) of zinc transporter encoding genes (** ***NtZIP1*** **and** ***NtHMA–A*** **) in transgenic tobacco lines and wild type plants under de–stress condition.**
(DOCX)
