# Supplementary figures and images for: The SbMT-2 Gene from a Halophyte Confers Abiotic Stress Tolerance and Modulates ROS Scavenging in Transgenic Tobacco
Source: PLoS One. 2014 Oct 23;9(10):e111379. doi: 10.1371/journal.pone.0111379 (PMC4207811; doi:10.1371/journal.pone.0111379)

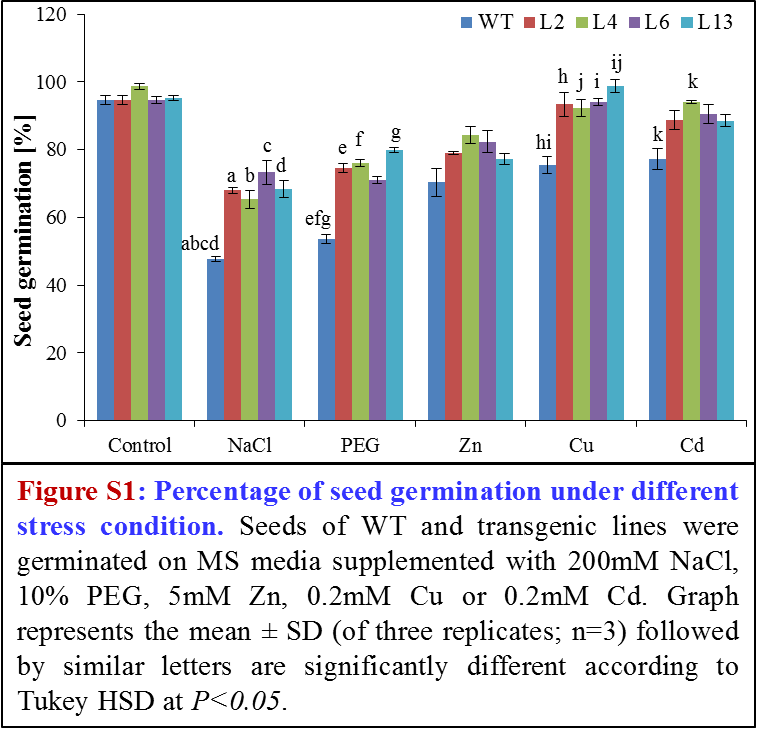

Supplement: Figure S1 — Percentage of seed germination under different stress condition. Seeds of WT and transgenic lines were germinated on MS media supplemented with 200 mM NaCl, 10% PEG, 5 mM Zn, 0.2 mM Cu or 0.2 mM Cd. Graph represents the mean ± SD (of three replicates; n = 3) followed by similar letters are significantly different according to Tukey HSD at P<0.05. (TIF) [file pone.0111379.s001.tif]

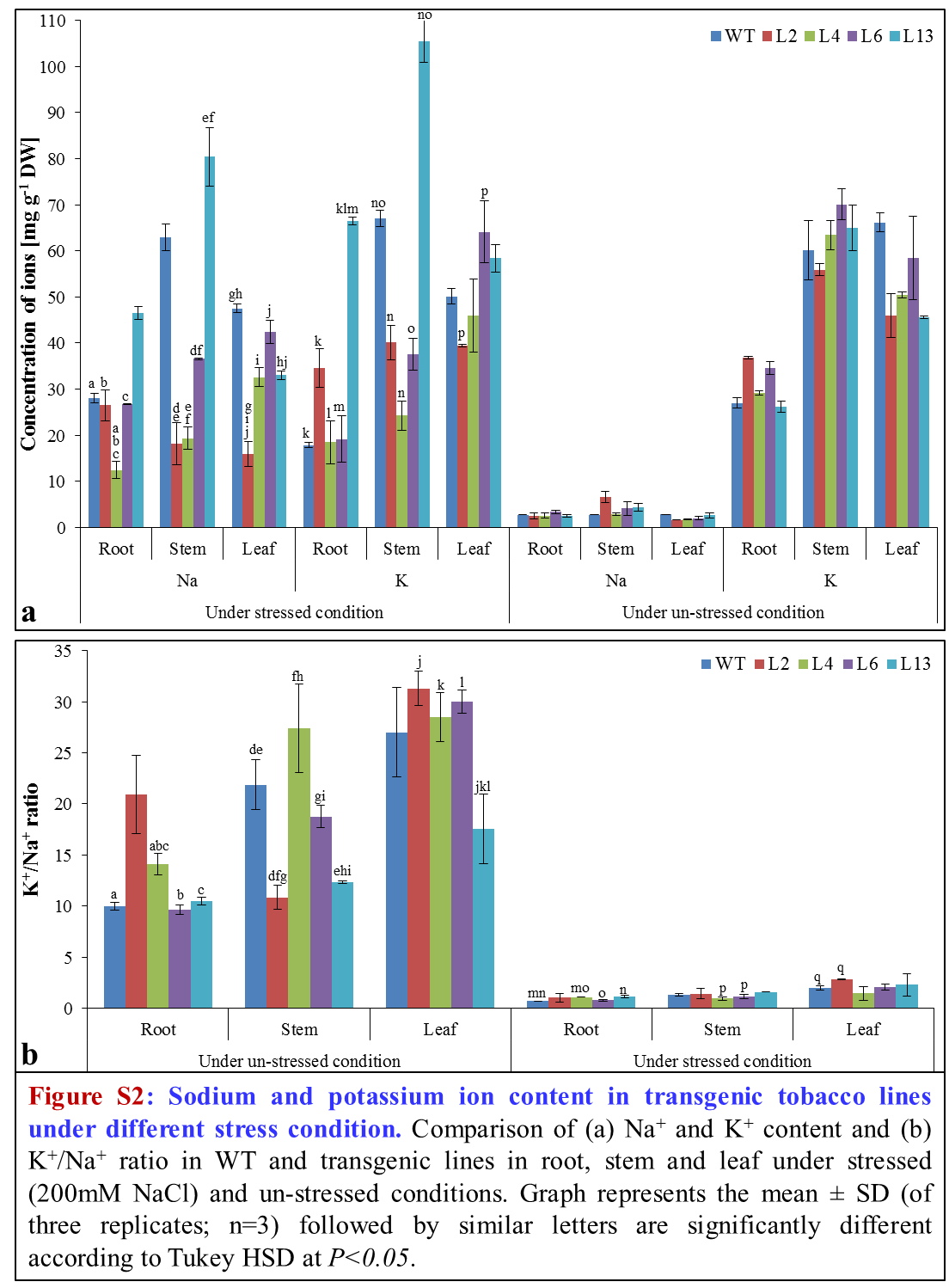

Supplement: Figure S2 — Sodium and potassium ion content in transgenic tobacco lines under different stress condition. Comparison of (a) Na+ and K+ content and (b) K+/Na+ ratio in WT and transgenic lines in root, stem and leaf under stressed (200 mM NaCl) and un-stressed conditions. Graph represents the mean ± SD (of three replicates; n = 3) followed by similar letters are significantly different according to Tukey HSD at P<0.05. (TIF) [file pone.0111379.s002.tif]
