# Supplementary material for: The SbMT-2 Gene from a Halophyte Confers Abiotic Stress Tolerance and Modulates ROS Scavenging in Transgenic Tobacco
Source: PLoS One. 2014 Oct 23;9(10):e111379. doi: 10.1371/journal.pone.0111379 (PMC4207811; doi:10.1371/journal.pone.0111379)
Supplement: Table S1 — Comparison of relative fold expression (down–regulation) of zinc transporter encoding genes ( NtZIP1 and NtHMA–A ) in transgenic tobacco lines and wild type plants under de–stress condition. (DOCX) [file pone.0111379.s003.docx]

**Table S1: Comparison of relative fold expression (down–regulation) of zinc transporter encoding genes (*NtZIP1* and *NtHMA–A*) in transgenic tobacco lines and wild type plants under de–stress condition.**

| **Genes/ lines** | **Zn** | **Cu** | **Cd** |
| --- | --- | --- | --- |
| ***NtZIP1*** |  |  |  |
| WT | –2.4 | –1.4 | –1.5 |
| L2 | –10.2 | –2.3 | –4.2 |
| L4 | –12.9 | –2.6 | –4.8 |
| L6 | –8.7 | –5.1 | –3.3 |
| L13 | –6.7 | –3.1 | –2.6 |
| ***NtHMA–A*** |  |  |  |
| WT | –1.1 | –1.2 | –1.1 |
| L2 | –1.5 | –2.2 | –1.8 |
| L4 | –1.6 | –1.3 | –1.9 |
| L6 | –1.9 | –1.0 | –1.3 |
| L13 | –1.8 | –1.4 | –1.2 |

To remove the metal stress, plants were re–cultured in 1/2 MS solutions for 48 h after the stress treatment
